# Characterization of SCF-Complex during Bovine Preimplantation Development

**DOI:** 10.1371/journal.pone.0147096

**Published:** 2016-01-29

**Authors:** Veronika Benesova, Veronika Kinterova, Jiri Kanka, Tereza Toralova

**Affiliations:** 1 Laboratory of Developmental Biology, Institute of Animal Physiology and Genetics Academy of Science of Czech Republic, v.v.i., Libechov, Czech Republic; 2 Faculty of Science, Charles University in Prague, Prague, Czech Republic; 3 Department of Veterinary Sciences, Czech University of Life Sciences in Prague, Prague, Czech Republic; IISER-TVM, INDIA

## Abstract

The degradation of maternal proteins is one of the most important events during early development, and it is presumed to be essential for embryonic genome activation (EGA), but the precise mechanism is still not known. It is thought that a large proportion of the degradation of maternal proteins is mediated by the ubiquitin-proteolytic system. In this study we focused on the expression of the Skp1-Cullin1-F-box (SCF) complex, a modular RING-type E3 ubiquitin-ligase, during bovine preimplantation development. The complex consists of three invariable components—Cul1, Skp1, Rbx1 and F-box protein, which determines the substrate specificity. The protein level and mRNA expression of all three invariable members were determined. *Cul1* and *Skp1* mRNA synthesis was activated at early embryonic stages, at the 4c and early 8c stage, respectively, which suggests that these transcripts are necessary for preparing the embryo for EGA. CUL1 protein level increased from MII to the morula stage, with a significant difference between MII and L8c, and between MII and the morula. The CUL1 protein was localized primarily to nuclei and to a lesser extent to the cytoplasm, with a lower signal in the inner cell mass (ICM) compared to the trophectoderm (TE) at the blastocyst stage. The level of SKP1 protein significantly increased from MII oocytes to 4c embryos, but then significantly decreased again. The localization of the SKP1 protein was analysed throughout the cell and similarly to CUL1 at the blastocyst stage, the staining was less intensive in the ICM. There were no statistical differences in RBX1 protein level and localization. The active SCF-complex, which is determined by the interaction of Cul1 and Skp1, was found throughout the whole embryo during preimplantation development, but there was a difference at the blastocyst stage, which exhibits a much stronger signal in the TE than in the ICM. These results suggest that all these genes could play an important role during preimplantation development. This paper reveals comprehensive expression profile, the basic but important knowledge necessary for further studying.

## Introduction

At the very beginning of early embryogenesis, all the mRNAs and proteins are of maternal origin and development is driven by these maternal reserves. As development proceeds, these reserves are stepwise degraded and the embryo takes over control of development. The degradation of maternal mRNAs is a gradual process, which peaks around the major wave of embryonic genome activation (EGA), and results in the degradation of the vast majority of maternal mRNAs at this time [1]. However, little information about protein degradation is available. Presumably, the degradation of maternal protein is essential for the start of embryonic genome transcription, but the precise mechanism of EGA initiation is still not known. The process is certainly not as rapid as mRNA degradation, and some of the proteins remain even after EGA [2–4]. It is thought that a large proportion of the degradation of maternal proteins is mediated by the ubiquitin-proteolytic system [5–8]. The ubiquitin-proteasome pathway directs protein degradation, and thus serves as a regulatory mechanism of various cellular processes. The ubiquitination of proteins is a stepwise process, which is managed by the cooperation of three enzyme complexes: E1 –ubiquitin activating enzyme; E2 –ubiquitin-conjugating enzyme and E3 –ubiquitin ligase [9]. The E3 ligases transfer ubiquitin from E2 to the substrate protein, and their plurality (caused by F-box protein variability) enables the specific labelling of various proteins.

In this study we have focused on the expression of the Skp1-Cullin1-F-box (SCF) complex, a modular RING-type E3 ubiquitin-ligase, in bovine preimplantation development. It is thought that up to 20% of ubiquitinated proteins are triggered for degradation by the SCF complex [10]. The complex consists of three invariable components—Skp1, Cullin 1 and Rbx1—and one of many F-box proteins, which determines the substrate specificity [11–13]. The deregulation of SCF-complex activity is included in the ethiopathogenesis of many diseases, including cancer [14,15].

### Invariant members of the SCF complex

#### Cullin 1

Cullin 1 is the backbone of the SCF complex and determines its activity. It forms two mutually exclusive complexes whose generation is based on neddylation (modification with the small ubiquitin-like protein Nedd8). When deneddylated, Cullin 1 binds to CAND1 (Cullin-associated and neddylation dissociated 1) and becomes ubiquitination inactive. After neddylation, CAND1 dissociates and Cullin 1 binds to Skp1 and forms the SCFcomplex, which enables protein ubiquitination [16,17].

Cullin 1, as an essential part of this E3 ubiquitin ligase, plays an important role in a large number of biological processes, such as cell cycle regulation, signal transduction, transcription or translation [18–20]. Two detailed studies concerning the role of cullin 1 in early mammalian embryogenesis were published in 1999 [21,22]. However, to the best of our knowledge, no major papers have been published since then. Both Delay and Wang found that Cullin 1 plays a crucial role during embryogenesis before the onset of gastrulation in mice [21,22]. However, its function during preimplantation development has not been specified. In *C*. *elegans*, cullin 1 likely participates in the degradation of proteins that directs the transformation of an oocyte into a rapidly developing, totipotent embryo [8]. It may play a role in hatching and post-hatching pre-attachment processes, as the expression of its mRNA significantly increases from a blastocyst to a hatched blastocyst [23]. Higher expression was shown to indicate a high quality embryo [24]. Cullin 1 also plays an important role in trophoblast cell invasion and placenta development [25].

We have recently found that cullin 1 is expressed from two different genes, both of them located on chromosome 4, only in distinct regions [26]. One of them was identified as Cullin 1 (NM_001193233) and was expressed from the late 8-cell stage to the blastocyst stage. Because of its expression, we also call this variant embryonic cullin 1. The other variant shared 83% homology and was expressed from the MII oocyte until the early 8-cell stage. The second variant was called cullin 1-like or maternal cullin 1. In somatic cells, embryonic cullin 1 is expressed.

#### Skp1 (S-phase kinase-associated protein 1)

Skp1 is a stable protein whose best-known functions are connected to the SCF complex [27] Skp1 bounds the catalytic core of the SCF complex to the F-box motif of the F-box protein [11–13]. It participates in the regulation of SCF complex deactivation by the deneddylation of cullin. Altered expression of Skp1 plays a role in the development of Parkinson´s disease [28,29] and lymphomas [30].

#### Rbx1 (ring-box 1; ROC1—regulator of cullins-1)

Rbx1 (together with Cullin 1) forms the catalytic core of the SCF complex. In addition to cullin 1, Rbx1 interacts with all six of the other cullins, and thus also activates other E3 ubiquitin ligases [31,32]. Rbx1 contains the RING finger domain that recruits the E2 ubiquitination enzyme and mediates the neddylation of cullin [33]. Rbx1 plays an unquestionable role in the embryogenesis of *C*. *elegans*, *D*. *melanogaster* and even mice, as silencing its mRNA causes embryonic death in these species [34–37]. It is also responsible for the normal progress of mouse and *Xenopus* oocyte meiosis [38–40] Rbx1 RNA silencing causes DNA double strand breaks, G2 arrest and aneuploidy [41], and is overexpressed in many cancers.

Nevertheless, little information is available on the role of the SCF-complex in early embryogenesis. The activity of the SCF complex during mammalian preimplantation development and the expression of each invariant component have not been described to date. In this paper, we describe the expression profile of the mRNA and protein of each component in detail. Furthermore, we define the SCF complex activity from the MII oocyte to blastocyst stage. The preimplantation development of the selected model organism, bovines, is highly similar to humans and other non-rodent mammals in terms of the timing of embryonic genome activation, epigenetic reprogramming, duration of the development and presence of the piRNA and Piwi proteins pool [42]. A similar expression profile can be hence expected in other mammals.

## Materials and Methods

### IVF and embryo culture

Unless otherwise indicated, the chemicals were purchased from Sigma (Sigma-Aldrich, St. Louis, MO) and plastic from Nunclon (Nunc, Roskilde, Denmark). Bovine embryos were obtained after the *in vitro* maturation of oocytes and their subsequent fertilisation and *in vitro* culture. Briefly, abattoir-derived ovaries from cows and heifers were collected and transported in thermocontainers in sterile saline at about 33°C. The cattle had been slaughtered (Jatky Rosovice, spol. s.r.o.; Slaughterhouse Rosovice) for the public edible meat. Those ovaries were discarded without any utilization. Hence, an ethics statement in our paper was not required. The follicles with a diameter between 5 and 9 mm were dissected with fine scissors and then punctured. The cumulus-oocyte complexes were evaluated and selected according to the morphology of the cumulus and subjected to in vitro maturation in TCM 199 (Earle´s salt) supplemented with 20 mM sodium pyruvate, 50 U/ml penicillin, 50 μg/ml streptomycin, 10% oestrus cow serum (ECS) and gonadotropins (P. G. 600, 15 U/ml; Intervet, Boxmeer, Holland) without a paraffin overlay in four-well dishes under a humidified atmosphere for 24 h at 39°C with 5% CO_2_.

For IVF, the cumulus-oocyte complexes were washed four times in PBS and once in Tyrode´s albumin lactate pyruvate (TALP) fertilisation medium, and transferred in groups of up to 30 to four-well dishes containing 250 μl TALP per well. The TALP medium contained 1.5 mg/ml BSA, 30 μg/ml heparin, 0.25 mM sodium pyruvate, 10 mM lactate and 20 μM penicillamine. One straw with frozen semen from one bull previously tested in the IVF system was thawed in a 40°C water bath, diluted with 2 ml TALP and centrifuged at 3500 g for 10 min. The spermatozoa were layered under 5×1ml TALP. The supernatant with the motile spermatozoa was isolated after 1h of swim-up at 39°C [43]. Spermatozoa were counted in a haemocytometer and diluted in the appropriate volume of TALP to give a concentration of 2×10^6^ spermatozoa/ml. A 250 μl aliquot of this suspension was added to each fertilisation well to obtain a final concentration of 1×10^6^ spermatozoa/ml. Plates were incubated under a humidified atmosphere with 5% CO_2_−5% O_2_−90% N_2_ for 20 h at 39°C.

At approximately 20 h post fertilisation (hpf), presumed zygotes were denuded by gentle pipetting and transferred to Menezo B2 medium (Veterinary Research Institute, Brno, Czech Republic) supplemented with 10% ECS and cultured in a humidified atmosphere of 5% CO_2_−5% O_2_−90% N_2_ (25 zygotes in 25 μl medium under liquid paraffin (Origio, Malov, Denmark)). The dishes were examined at 24 h post isolation and 34, 44, 72, 96, 120, 156 and 180 hpf, and MII oocytes and two-cell, four-cell, early eight-cell, late eight-cell, morula, blastocysts and hatched blastocysts were collected at each time point respectively.

### Quantification of mRNA expression

Poly (A)+ mRNA was extracted from the pools of 20 oocytes and embryos at each stage of development, using a Dynabeads mRNA DIRECT Micro Kit (Invitrogen Dynal AS, Eugene, OR) according to the manufacturer´s instructions. Before isolation, 1 pg of Luciferase mRNA (Promega Madison, WI) per oocyte/embryo was added as an external standard. Primer sequences were designed using Beacon Designer 7 from the bovine cullin 1-like, cullin 1, Skp1 and Rbx1 gene sequences (GeneBank accession numbers XM_589507.3, NM_001193233.1, NM_001034781, NM_001034781) (Table 1).

**Table 1:** Primer details.

Primer	Sequences	Annealing temperature (°C)	Amplicon size (bp)
*Cul 1-like*	5´- CGG ACT GGA GCC AGA ATC CCA—3´	60	178
(XM_589507.3)	5´- GTC TGG GCT TGA GGG GAC ACA– 3´		
*Cul1*	5´- AAC CCC CAC GGA CTC AAG CAG A– 3´	60	173
(NM_001193233.1)	5´- GCC CCT CGA GCT TGG TTT GAC T– 3´		
*Skp1*	5´- GCC ATC TCC TTG AGC CCT AC– 3´	55	172
(NM_001034781)	5´- CAT TTG GCA AGG GGA CTG GA– 3´		
*Rbx1*	5´- CAG GCG TCC GCT ACT TCT G– 3´	63	93
(NM_001034781)	5´- TGT TTT GAG CCA GCG AGA GA– 3´		
Luciferase	5´- ACT TCG AAA TGT CCG TTC GG– 3´	55	633
	5´- ACT TCG AAA TGT CCG TTC GG– 3´		

The expression of specific mRNA was measured by quantitative RT-PCR. mRNA was amplified using a OneStep RT-PCR kit (Qiagen, Hilden, Germany) with real-time detection using SybrGreen fluorescent dye. The reaction composition was Qiagen OneStep RT-PCR buffer (1×), dNTP Mix (400 μM of each), forward and reverse primers (both 400 μM), SybrGreen (1:50 000 of 1000× stock solution; Invitrogen), RNasin Ribonuclease Inhibitor (Promega; 0.2 μl), QIAGEN OneStep Enzyme Mix (0.5 μl), and template RNA. Reaction conditions were as follow: RT at 50°C for 30 min, initial activation at 95°C for 15 min, cycling: denaturation at 94°C for 15 s, annealing at 60°C for 20 s (Cul 1-like, Cul1), at 55°C for 20 s (Skp 1), at 63°C for 20 s (Rbx 1), and extension at 72°C for 30 s. The final extension step was held for 10 min at 72°C. The real-time RT-PCRs were run in duplicate, with all samples (oocytes and all embryos stages) in the same reaction. The experiments were carried out in a RotorGene 3000 (Corbett Research, Morthlake, Australia). Fluorescence data were acquired at 3°C below the melting temperature to distinguish the possible primer dimers.

The relative concentration of the template in the different samples was determined using comparative quantification in analysis software (Corbett Research) as described in [44]. The results were normalised according to the relative concentration of the external standard (Luciferase). The take-off points were calculated as 20% of the second-derivative maximum level (RotorGene 3000 operation manual; Corbett Research). Products were verified by melting analysis and gel electrophoresis on 1.5% agarose gel with ethidium bromide staining. Experiments were repeated at least four times.

### Alpha-amanitin treatment

To block RNA polymerase II-dependent transcription, α-amanitin (Sigma-Aldrich, St. Louis, MO) was added to the culture medium at a final concentration of 100 μg/ml for either from the one-cell stage to two-cell stage (20–34 hpf), from the one-cell stage to four-cell stage (20–44 hpf), from the four-cell stage to early eight-cell stage (44–72 hpf) or from the four-cell stage to late eight-cell stage (44–96 hpf). After the α-amanitin treatment, the embryos were washed with PBS, immediately frozen and stored at -80°C. Control embryos were collected at the same time interval as their treated counterparts from the same fertilization/cultivation group, washed with PBS, immediately frozen and stored at -80°C. All pools were done in triplicate and contained 20 embryos.

### Immunofluorescence

Embryos were fixed in 4% paraformaldehyde for 50 min at 4°C. Fixed embryos were processed immediately or stored in PBS for up to 3 weeks at 4°C. After washing in PBS, the embryos were incubated in 0.5% (v/v) TritonX-100 for 15 min. All subsequent steps were done in PBS supplemented with 0.3% (w/v) BSA and 0.05% (w/v) saponin (PBS/BSA/sap). Embryos were blocked with 2% normal goat serum (NGS; Millipore Biosciences; St. Charles, MO) for 1 h and incubated with rabbit anti-cullin 1 antibody (Abgent AJ 1205a, San Diego, CA) 1:100, rabbit anti- ROC1 (Abcam, Cambridge, UK) 1:100 or mouse anti-SKP 1 (Abcam 4E11, Cambridge, UK) 1:100 in PBS/BSA/sap overnight at 4°C. After thorough washing, the embryos were incubated with goat anti-rabbit antibody conjugated with FITC 1:350 (Santa Cruz Biotechnology, Santa Cruz, TX) or goat anti-mouse conjugated with Alexa Fluor 594 1:800 (Invitrogen, Eugene, OR) in PBS/BSA/sap for 1 h at room temperature in the dark. After washing, the nuclei were stained and the embryos were mounted with Vectashield HardSet Mounting Medium with DAPI (Vector Laboratories, Peterborough, UK). Controls of immunostaining specificity were carried out by omitting the primary antibody or using another species-specific secondary antibody conjugate.

### In situ Proximity ligation assay (PLA)

To test the activity of the SCF complex, the interaction of CUL1 and SKP1 was localized and quantified by Duolink (Olink Bioscience, Uppsala, Sweden). Unless otherwise indicated, chemicals were part of the Duolink. Embryos were fixed and permeabilized in the same way as for immunofluorescence staining. The Duolink in situ proximity ligation assay (PLA) was performed according to the manufacturer´s instructions. Embryos were blocked with 2% normal donkey serum (NDS; Santa Cruz Biotechnology, Santa Cruz, TX) in PBS/BSA/sap. Then they were incubated with rabbit anti-cullin 1 antibody (Abgent AJ 1205a, San Diego, CA) 1:100 together with mouse anti-SKP 1 antibody (Abcam 4E11, Cambridge, UK) 1:100, overnight at 4°C. After thorough washing, the embryos were cultivated with the PLA probes, diluted in the antibody diluent buffer, for 1 h at 37°C. After washing, the ligation solution was added for 30 min at 37°C. After washing in washing buffer A, the amplification stock (5x amplification stock, polymerase, RNase-free water) was added to the embryos for 100 min at 37°C in the dark. After washing, the nuclei were stained and the embryos were mounted with Vectashield HardSet Mounting Medium with DAPI (Vector Laboratories, Peterborough, UK). Controls of the assay were prepared by omitting either the probe or primary antibody.

The samples were examined with a Leica SP 5 (Leica Microsystems AG, Wetzlar, Germany). The images were processed using the software Fiji (http://fiji.sc).

### Western blotting

Unless otherwise indicated, chemicals were purchased from Sigma. Embryos and oocytes (25 per extract) were lysed in 15 μl of Blue Loading Buffer (772, Cell Signaling Technology, Danvers, MA) with dithiothreitol, boiled for 5 min and subjected to 12% SDS-PAGE. Proteins were transferred from gels to an Immobilon P membrane (Millipore Biosciences, Billerica, MA) using a semidry blotting system (Whatman Biometra GmbH, Hoettingen, Germany) for 28 min at 5 mA/cm^2^. The blocking of the membrane was performed in 3% BSA in TBS-Tween buffer (TBS-T, 20 mM Tris, pH 7.4, 137 mM NaCl and 0.5% Tween 20) for cullin 1 and RBX1, and in 5% non-fat milk in TBS-T for SKP1, for 1 h and incubated overnight with rabbit anti-cullin 1 (Abgent 1205a) 1:1000, rabbit anti-ROC 1 (Abcam) 1:1000 or mouse anti-SKP 1 antibody (Abcam 4E11) 1:1000 in 5% non-fat milk/TBS-T. The results of the SKP 1 antibody were verified with a different mouse anti-SKP1 antibody (Abcam 1H9) 1:1000. After washing in TBS-T, the membranes were incubated with HRP-conjugated donkey anti-rabbit or donkey anti-mouse IgG antibody (both 1:7500; Jackson Immuno Research, Suffolk, UK) in 5% non-fat milk/TBS-T for 1 h at room temperature. Proteins were visualized with Luminata Crescendo Western HRP (Merck Millipore, Darmstadt, Germany). The data were processed using Quantity One software (Bio-Rad).

### Statistical analysis

The data were analysed using SigmaStat 3.0 software (Jandel Scientific, San Rafael, CA). The One-Way ANOVA test or Holm-Sidak method were used. P≤0.05 was considered statistically significant.

## Results

### The mRNA expression of invariant members of SCF complex

**Fig 1:**
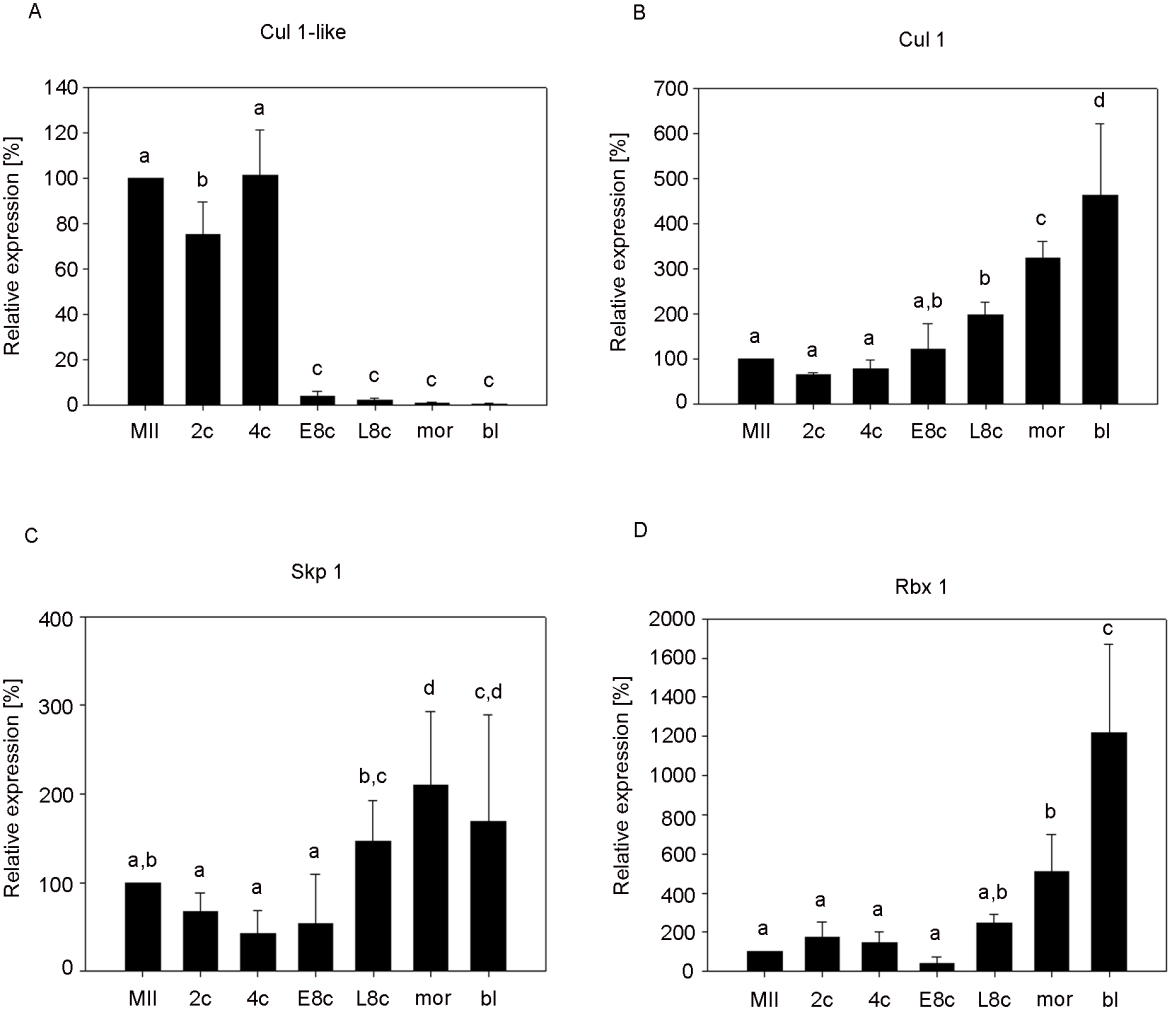
Relative mRNA expression of invariant members of SCF complex. Untreated embryos. The data were normalised according to the relative concentration of the external standard (luciferase mRNA, 1 pg per oocyte/embryo). (A) *Cul 1-like*, (B) *Cul1*, (C) *Skp1*, (D) *Rbx1*. Bars show ± S.D. ^a,b,c,d^. Values with different superscripts indicate statistical significance (P<0.05). (MII, MII stage oocyte; 2c, two-cell stage embryo; 4c, four-cell stage embryo; E8c, early eight-cell embryo; L8c, late eight-cell stage; mor, morula; bl, blastocyst).

By quantitative RT-PCR analysis with variant-specific primers, both *Cul 1-like* and *Cul1* transcripts were detected in bovine embryos before and after embryonic genome activation (EGA). The expression pattern of the maternal *Cul 1-like* transcript during the in vitro culture (from MII oocyte to blastocyst stage) exhibited a relatively constant high level from MII to 4c, however at the early 8c stage (72 hours post fertilization, hpf), mRNA level rapidly decreased (p<0,001) and continued decreasing until the blastocyst stage (Fig 1A). Inversely, the level of *Cul1* mRNA was low from MII to 4c, then rose slightly up to the early 8c (72 hpf), and significantly increased at the late 8c stage (96 hpf) (p<0.05). The level was highest at the blastocyst stage (Fig 1B).

The *Skp1* mRNA level was similar to *Cul1* during bovine preimplantation development. The transcript level slightly decreased from MII to 4c and started to rise at early 8c (72 hpf). A significant increase occurred at late 8c (p<0.05) and rose further up until the morula stage. Transcript nonsignificantly decreased at the blastocyst stage (Fig 1C).

The *Rbx1* mRNA was stable from MII to 4c, the level fell at early 8c but increased after EGA, at late 8c, and rose rapidly from the morula to blastocyst stage (p<0.001) (Fig 1D).

**Fig 2:**
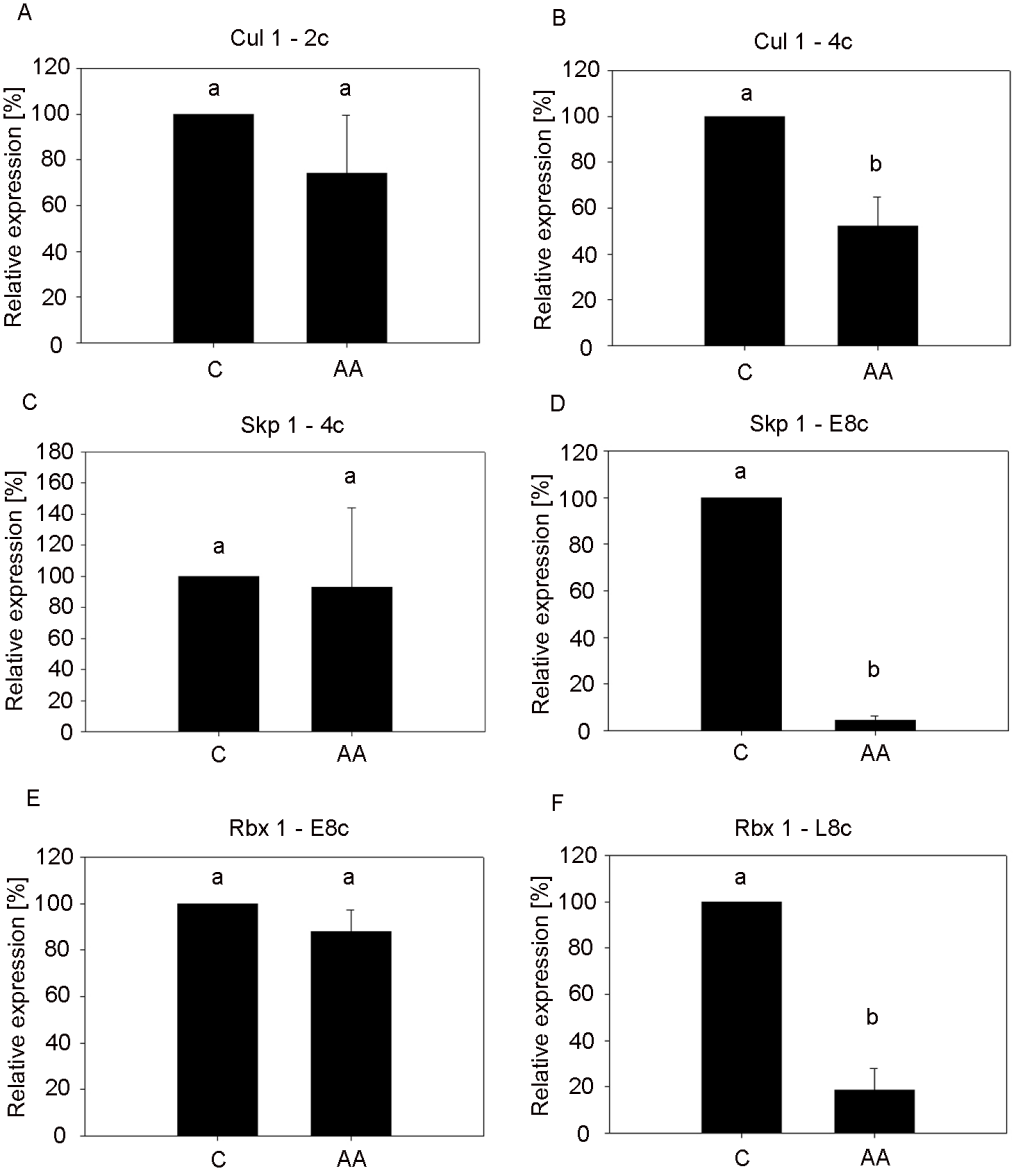
Relative mRNA expression of invariant members of SCF complex, embryos treated with α-amanitin. The data were normalised according to the relative concentration of the external standard (luciferase mRNA, 1pg per embryo). (A) *Cul1* –embryos treated from one-cell to two-cell stage, (B) *Cul1* –embryos treated from one-cell to four-cell stage, (C) *Skp1 –*embryos treated from one-cell to four-cell stage, (D) *Skp1* –embryos treated from four-cell to early eight-cell stage, (E) *Rbx1* –embryos treated from four-cell to early eight-cell stage, (F) *Rbx1* –embryos treated from four-cell to late eight-cell stage. Bars show ± S.D. ^a,b^ Values with different superscripts indicate statistical significance (P<0.05). (C, control group of untreated embryos; AA, group of embryos treated with α-amanitin; 2c, two-cell stage embryo; 4c, four-cell stage embryo; E8c, early eight-stage embryo; L8c, late eight-cell stage embryo).

To determine at which stage the transcription of *Cul1* from the embryonic genome begins, embryos were cultured in the presence of α-amanitin, an RNA II polymerase inhibitor. There was a significant (p<0.001) decrease in *Cul1* and *Skp1* at the 4c and early 8c stage (Fig 2A–2D), respectively, indicating that their transcription is initiated prior to major embryonic genome activation (EGA). On the other hand, the significant difference between the control group and α-amanitin-treated group in *Rbx1* occurred at the late 8c stage (Fig 2E–2F), at the time of the EGA. These results show the importance of the invariant members of the SCF complex in preimplantation development. The impact of α-amanitin treatment on transcript level at the different stages was also examined (S1 Fig).

### Protein expression and localization throughout bovine preimplantation development

#### Cullin 1

**Fig 3:**
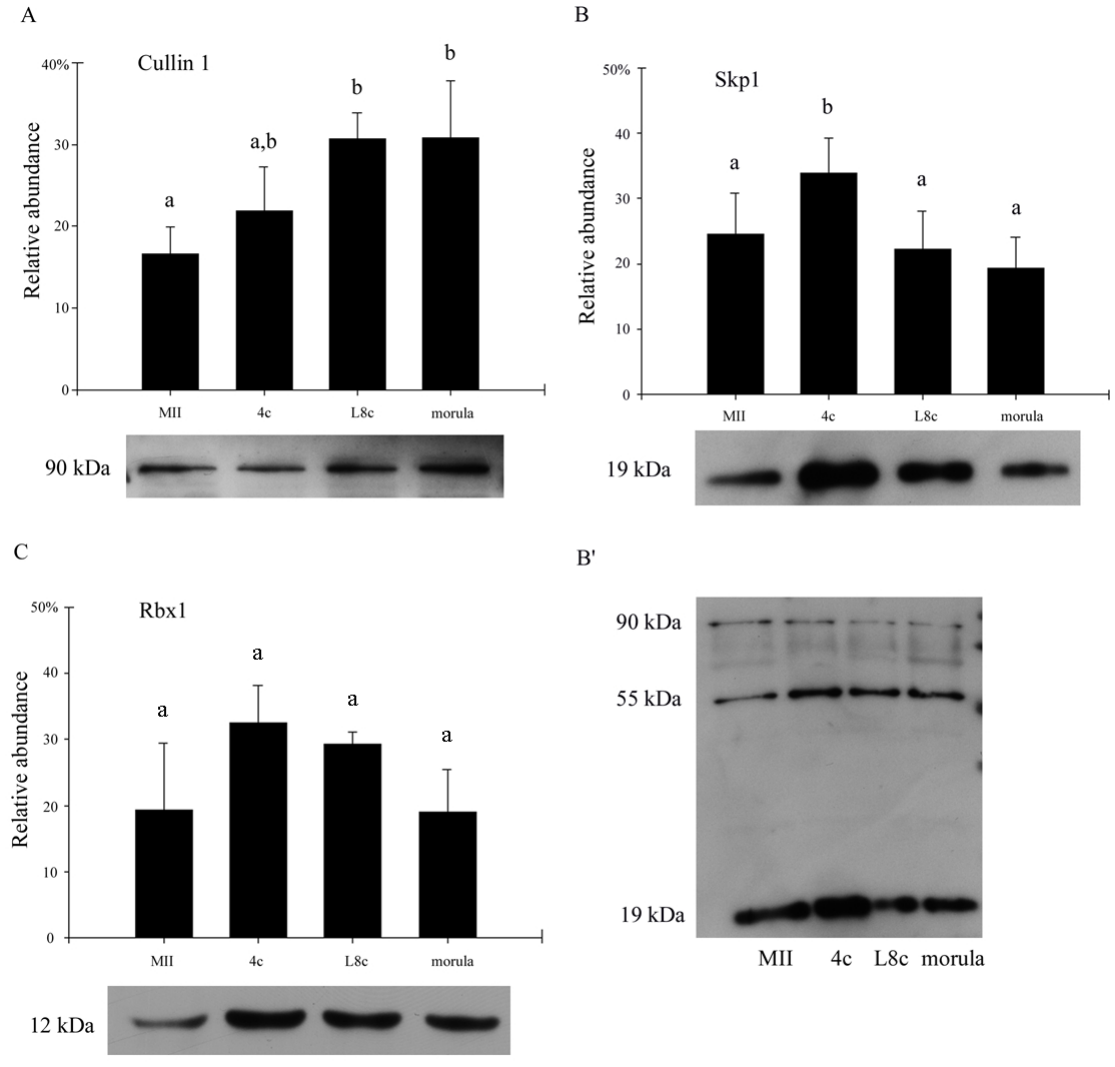
Quantification of protein level after western blot analysis of bovine oocytes and preimplantation embryos. 30 embryos per lane. A) Cullin 1; B) SKP1 (antibody 4E11); C) RBX1. The data were processed using Quantity One software (Bio-Rad). 100% represents the sum of the trace quantities of all bands; relative abundance (y-axis) represents the percentage of each band. Bars show mean ± S.D. ^a,b^Values with different superscripts indicate statistical significance (P<0.05). The experiments were repeated at least three times, and representative western blot images are shown below the graph. B’) Representative image of additional bands (approximate size 55 and 90 kDa). (MII, MII oocytes; L8c, late eight-cell stage embryos; 4c, four-cell stage embryos).

The level of cullin 1 protein gradually increased from MII oocytes (MII) to morula-stage embryos with a significant increase in protein level from the MII to late 8-cell stage and morula stage (p<0.01) (Fig 3A).

**Fig 4:**
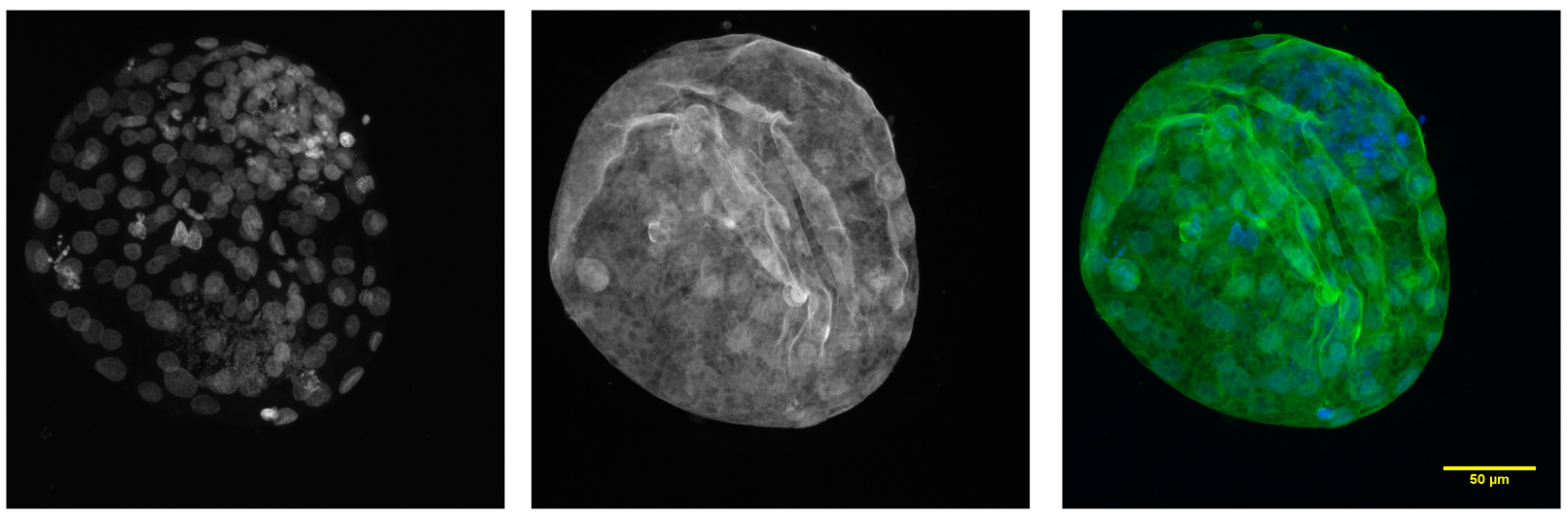
Confocal laser scanning microscopy of CUL1 of bovine blastocysts. Nuclei (DAPI)–blue; cullin 1 –green.

The localization of Cullin 1 was already described in our previous paper [26], thus here we present only the immunofluorescent staining of blastocysts. The protein was quite abundant throughout the whole embryo and localized primarily to nuclei and to a slightly lesser extent to the cytoplasm. However, we found a difference in the staining of the inner cell mass (ICM) and trophectoderm (TE). Even though Cullin 1 was present in both of them, the staining was noticeably less intensive in the ICM (Fig 4).

#### SKP1

The level of SKP1 protein significantly increased from MII oocytes to 4c embryos, but then significantly decreased again (p<0.05 in all cases). There were no statistically significant differences between MII oocytes, L8c embryos and morulas (p>0.05) (Fig 3B). In addition to the expected cca 19-kDa band, we detected two other weaker bands with approximate sizes of 55 and 90 kDa (Fig 3B’). This finding was verified using another SKP1-specific antibody (S2 Fig).

**Fig 5:**
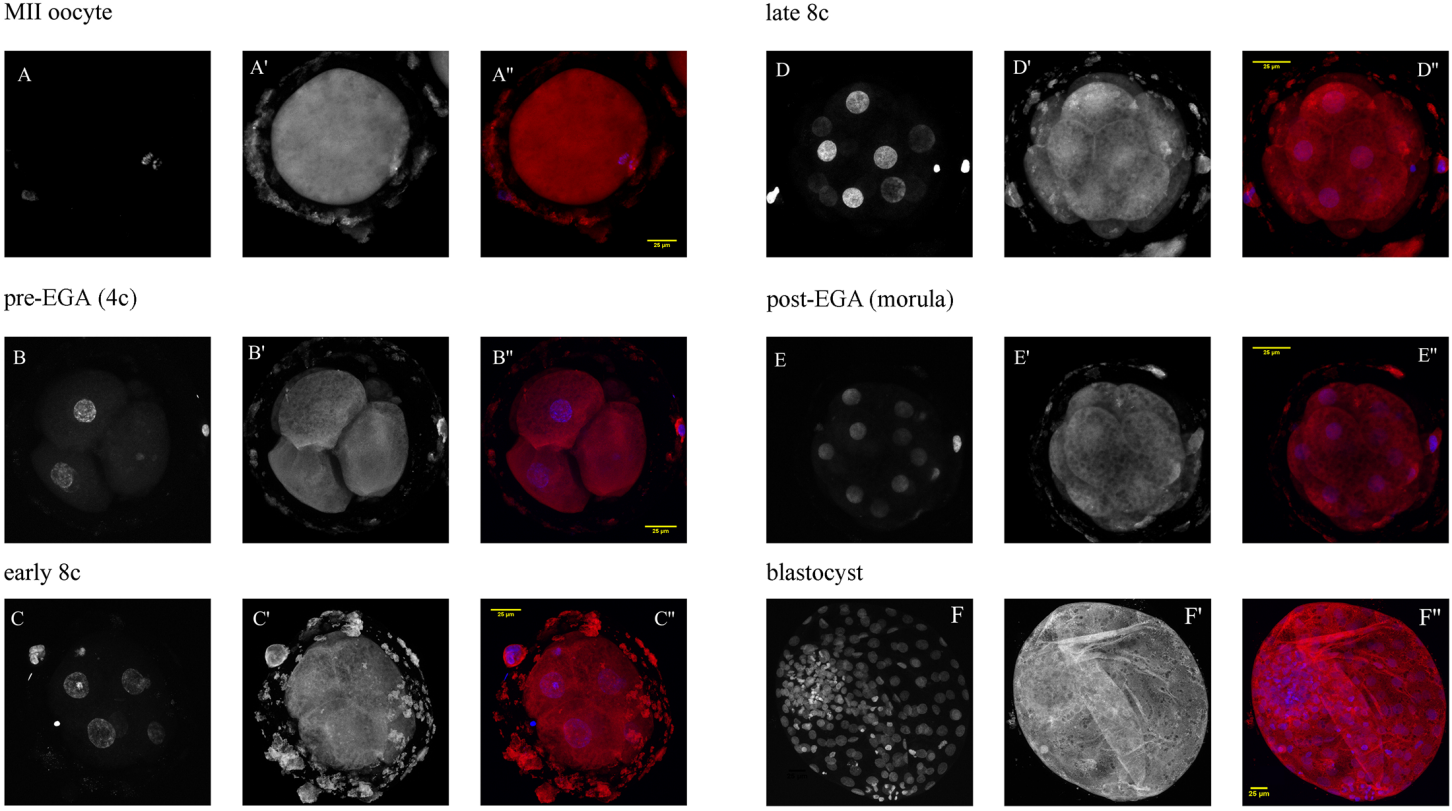
Confocal laser scanning microscopy of SKP1 from MII oocytes to blastocyst-stage embryos. The embryos were labelled with mouse monoclonal anti-SKP1 antibody (A’–F’) and the nuclei were stained with DAPI (A—F). In overlaid images (A”–F”), SKP1 is red and DNA blue.

The protein was distributed throughout the cell, and to some extent was also present at the zona pellucida. In MII oocytes, we found an accumulation of the immunofluorescence signal around the nucleus. In blastocysts, the staining was slightly less intensive in the ICM (Fig 5).

#### RBX1

**Fig 6:**
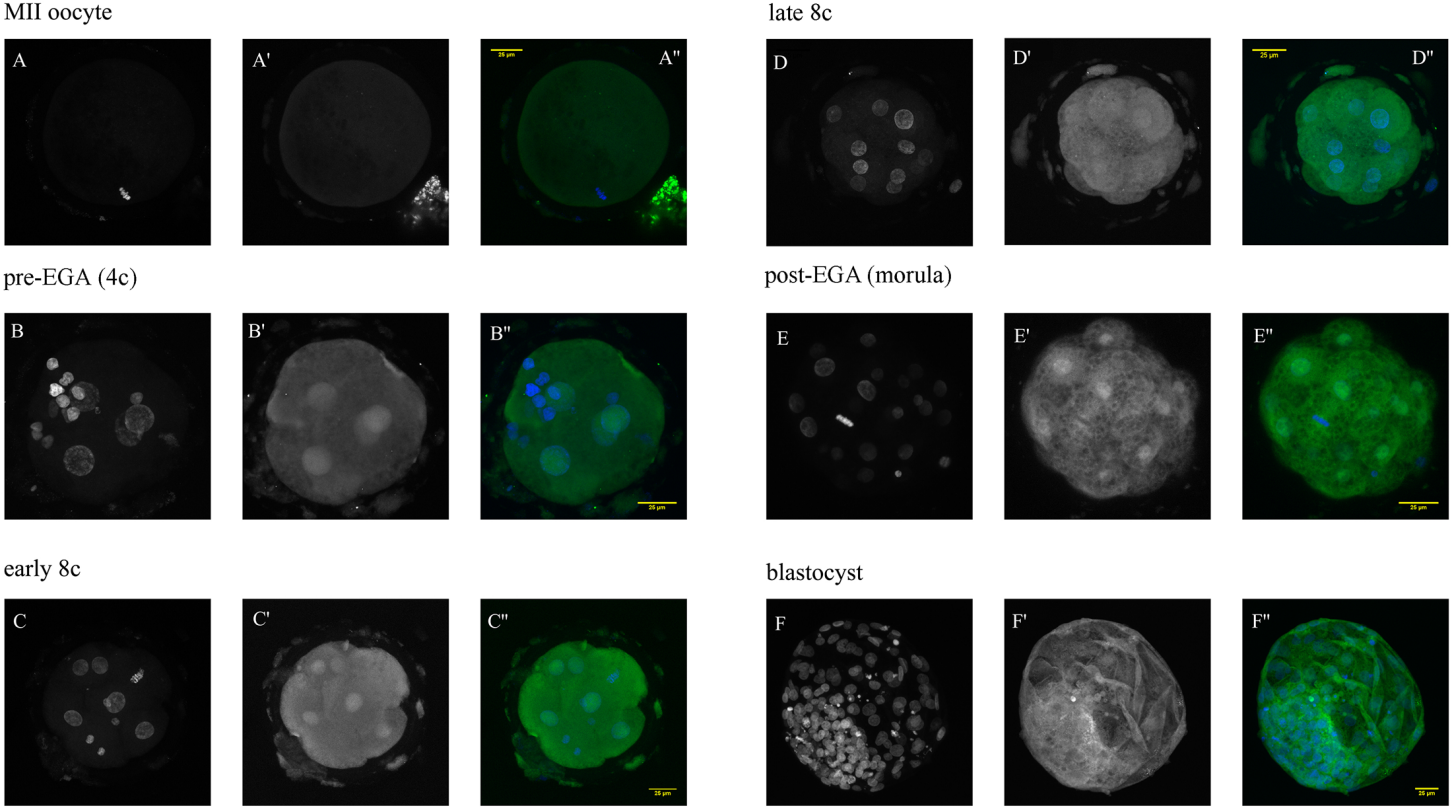
Confocal laser scanning microscopy of RBX1 from MII oocytes to blastocyst-stage embryos. The embryos were labelled with rabbit monoclonal anti-RBX1 antibody (A’—F’) and the nuclei were stained with DAPI (A–F). In overlaid images (A”–F”), RBX1 is green and DNA blue.

There were no statistically significant differences between the developmental stages (p>0.05) (Fig 3C). However, in MII oocytes and morulas, the protein was noticeably less abundant than in 4c and L8c. The protein was localized to the nucleus and cytoplasm at all developmental stages. At the blastocyst stage, the protein seems to be localized to the ICM and TE with the same intensity (Fig 6).

### SCF complex activity

**Fig 7:**
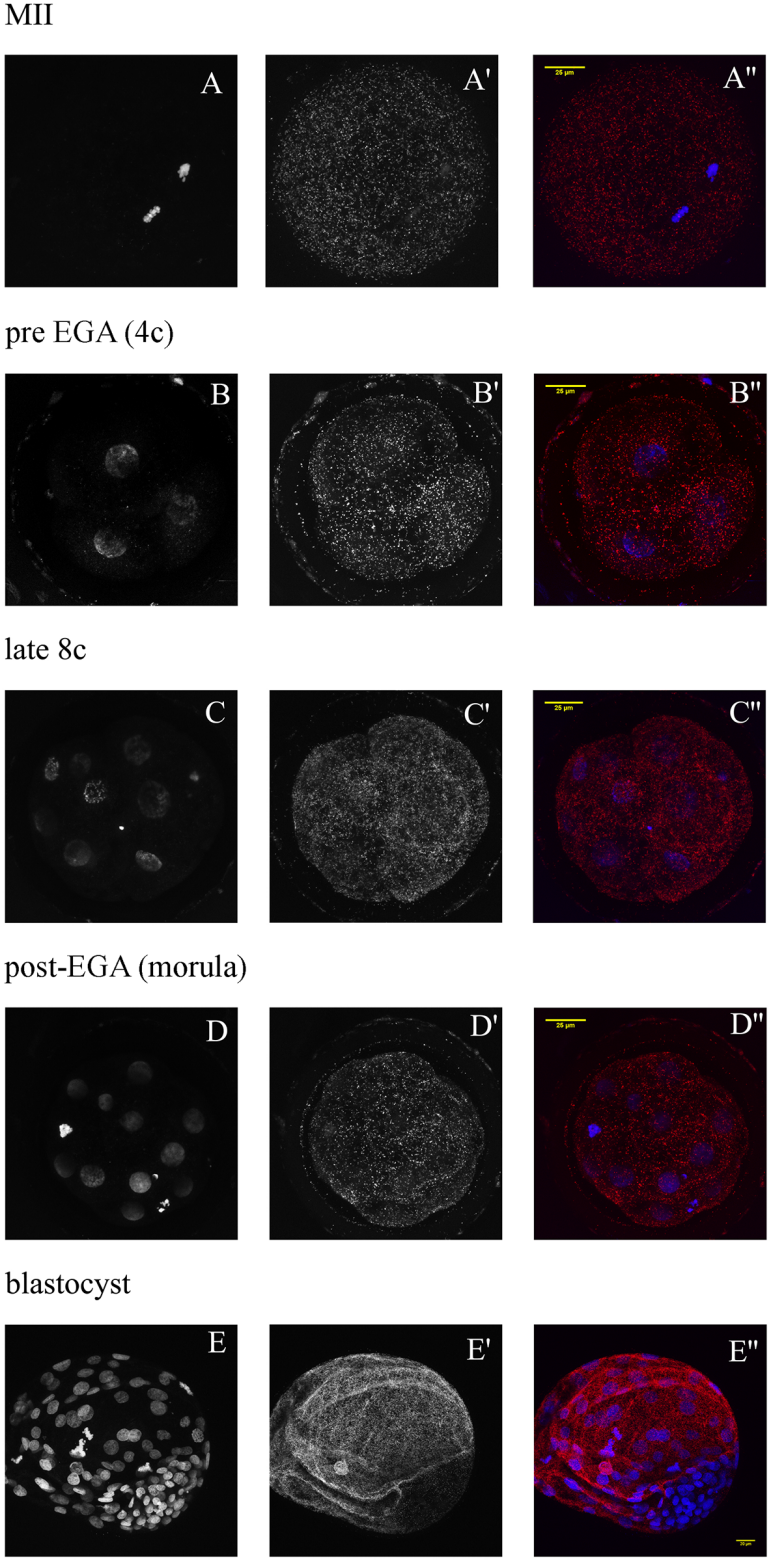
Confocal laser scanning microscopy of MII oocytes and preimplantation embryos after Duolink *in situ* PLA analysis. PLA signal indicates Cul1-Skp1 interaction (A’-E’) and the nuclei were stained with DAPI (A-E). In merges (A”-E”), PLA signal is red and DNA blue.

**Fig 8:**
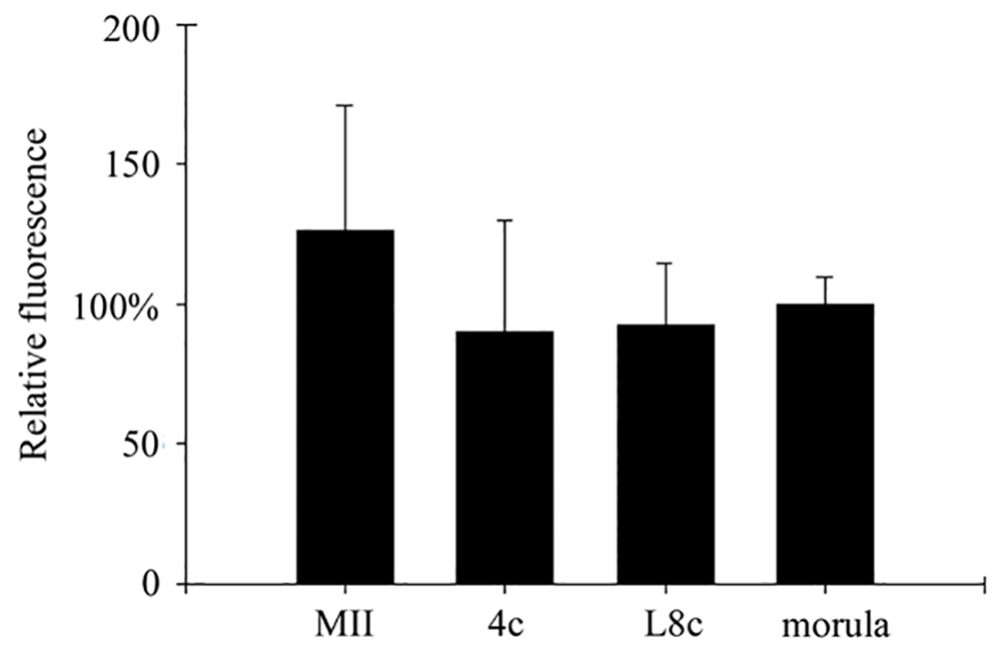
Relative fluorescence after analysis of Skp1/Cul1 interaction. The relative fluorescence (y-axis) represents the emitted fluorescence signal in a single embryo normalised to the mean of the measured fluorescence signal of morulas. There were in total 15 embryos per experimental group. The embryos were analysed using confocal scanning microscope in groups. Each group included an MII oocyte, a 4-cell stage embryo, late 8-cell stage embryo and morula. (MII, MII oocytes; L8c, late eight-cell stage embryos; 4c, four-cell stage embryos).

The interaction of Cullin 1 and Skp1, which indicates SCF complex activity, was monitored in groups consisting of MII oocytes, 4c embryos, late 8c embryos and morulas (Fig 7A–7D´´). The intensity of the fluorescence signal was measured and compared between these developmental stages. Blastocysts were analysed in a separate experiment. There was no statistically significant difference in PLA signal intensity between the groups; however, in MII oocytes the signal was noticeably more intensive than in all embryonic stages (Fig 8). At the blastocyst stage, there was almost no PLA signal in the ICM and on the other hand, the signal was very intensive in the TE (Fig 7E–7E´´).

## Discussion

The function of the SCF complex and its members in somatic cells, its necessity for oogenesis [39,45–48] spermatogenesis [49,50] and early development [8] and the need for precise regulation of proteolytic processes in early embryogenesis suggest the potential importance of the complex during mammalian preimplantation development.

To the best of our knowledge, the role of the SCF complex during this period has not been examined to date, nor has the expression profile of its members been determined. However, it is not possible to study the involvement of the SCF complex in preimplantation protein degradation without this knowledge. Thus, we determined thorough expression profiles of both the mRNAs and proteins of three invariant SCF complex members (Cullin 1, Rbx1, and Skp1). We also described the course of SCF complex activity throughout preimplantation development.

The dynamics of mRNA expression between individual stages reflects the importance of the gene during preimplantation development. Genes activated no later than during the major wave of EGA are assumed to be the most important. It was shown that silencing genes activated before or during EGA often results in early developmental arrest or substantially lowers its quality [51–53]. Genes related to protein ubiquitination are usually activated at the 8-cell stage [54]. However, we found earlier activation of two of the three invariant members of the SCF complex. The embryonic transcription of *Cul1* and *Skp1* starts in the 4c and early 8c stage, respectively, the transcription of *Rbx1* during a major wave of EGA (Fig 1). This is why the necessity of these genes and in fact the whole of the SCF complex for the normal course of EGA seems to be undisputed. The expression of Cullin 1 starts in the very early stages of embryogenesis, both in bovines and Drosophila [55]. Thus the embryonic expression of Cullin 1 seems to be the most important of the SCF complex members. This is further supported by the existence of the two Cullin 1 variants. These transcripts represent the products of two different genes, both present on chromosome 4 (UniGene IDs: maternal cullin 1—BT.36789; embryonic cullin 1—BT.6490) [26]. The transcripts share 83% identity, however the predicted protein of maternal cullin 1 shares only 78% identity with GenPept protein ID: NP_001180162.1 –cullin 1, *Bos taurus*. The maternal variant is probably a pseudogene raised by gene duplication [26] and is referred to as the *Bos taurus* cullin 1 pseudogene (LOC539792). Filippov [55] found that in *Drosophila* only the protein, not the transcript, is maternally inherited from the oocyte to embryo. This could be actually caused by the existence of two mRNA variants and non-detection of the maternal one. A similar expression change during preimplantation development was found in murine eIF-1A. In this case, the maternal and embryonic transcripts utilize different promoters, giving rise to diverse transcriptional products. In oocytes a TATA-containing promoter is used, while in embryos a TATA-less promoter is used [56]. The authors hypothesize that the increased utilization of the TATA-less promoter during EGA and further embryonic development can contribute to the smooth running of gene expression reprogramming, and is related to an increased expression of housekeeping genes or totipotent marker Oct4, which are both expressed from a TATA-less promoter. However both Cul1 variants are expressed from a TATA-less promoter, and thus the expression shift cannot be associated with different promoter utilization. Another similar case is the expression of the pseudogenes Zscan4d and Zscan4c in 2-cell stage murine embryos and murine embryonic stem cells, respectively [52]. However, in this case Zscan4 pseudogenes are not expressed in any other tissues or cell types, or maternally in the oocyte. Thus to the best of our knowledge, such a shift as in Cullin 1 variant expression has not been described elsewhere to date. Yet it seems that the activation of embryonic Cullin 1 transcription is important for normal preimplantation development, since it takes place as early as in the 4c stage. Even though at this stage the maternal variant was still predominant, almost no maternal cullin 1 was detected at the early 8c stage, and we can say that from this stage onwards the embryo is fully dependent on the embryonic variant.

Using Duolink in-situ PLA analysis, we monitored the interaction of Cullin 1 and Skp1, which indicates the activity of the SCF complex. The PLA signal was found in all developmental stages and was comparably intense in the examined embryonic stages. The highest activity was found in MII oocytes, where the proper degradation of protein is crucially important for normal maturation, however even this difference was not statistically significant. Generally, APC/C is thought to be the most important ubiquitin-ligase for meiotic maturation reviewed in [57], but it was found that the degradation of mitotic APC/C is controlled by the SCF complex [50]. Hence we hypothesize that the SCF complex also plays a similar role during meiosis. The PLA results indicates that the SCF complex is likely required for controlling the whole of preimplantation development, and that both maternal and embryonic variants of cullin 1 should be functional. However, since embryonic cullin 1 mRNA starts to be transcribed as early as the 4c stage, the shift seems to be required for preparing the embryo for EGA and it is still needed in further stages of preimplantation development. The level of cullin 1 protein gradually increased from MII oocytes (MII) to the morula stage. Since the localization of cullin 1 protein in bovine preimplantation embryos has been described in our previous paper [26], here we only present the data obtained in blastocysts. From the 2c to morula stage, the protein is dispersed throughout the blastomere and is present in the whole embryo [26]. Here we have found that at the blastocyst stage, cullin 1 tends to also localise to nuclei and it started to be predominantly localized in the trophectoderm rather than the inner cell mass. This is in accordance with the fact that Cullin 1 plays a key role in trophoblast cell invasion and placenta development [24]. An even greater difference was found in the staining intensity between TE and ICM after PLA staining. Almost no signal was detected in the ICM. These findings confirm the occurrence of ubiquitination in the trophectoderm [58]. Surprisingly, this activity did not correlate with the protein abundance of any of the three examined genes. Cullin 1 is thought to be the limiting factor for SCF complex activity [59], however the protein level increased gradually from MII oocytes to L8c-stage embryos, and then remained stable at the morula stage. The PLA activity was the highest in MII oocytes, nonsignificantly, though noticeably decreased in the 4c stage and remained stable until the morula stage. Thus both the localization of the proteins and the PLA signal at the blastocyst stage and the progress of protein level and SCF complex activity suggest that all three basic components of the SCF complex, including Cullin 1, also have other functions than merely being components of the SCF complex. This is especially obvious in Rbx1, which is known to interact with all other members of the cullin family, and thus is also part of other E3 ligases [30,60]. Both the mRNA and protein expression of Rbx1 defy the expression profiles of the other two invariant members of the SCF complex. Thus, even though Rbx1 is necessary for SCF complex functionality, and the complex is non-functional after Rbx1 silencing [40], RBX1 expression does not correlate with SCF complex activity. This applies to a lesser extent for the other two proteins, even though to the best of our knowledge all currently known functions of cullin 1 are related to the SCF complex, and Skp1 has a few functions besides the SCF complex in lower organisms [27,61]. We assume that the presumptive Skp1 complexes represented by the higher bands after western blot analysis are also not related to the SCF complex because of their size.

In conclusion, the mRNA and protein expression of the basic components of the SCF complex (Cullin 1, Skp1 and Rbx1) suggest that all these genes are essential for normal preimplantation development. The early activation of *Cul1* and *Skp1* mRNA expression suggests that these genes are necessary for preparing the embryo for EGA. SCF complex-mediated ubiquitination takes place at approximately the same level throughout the whole of preimplantation development. However at the blastocyst stage, the ubiquitination is concentrated specifically to the trophectoderm, the emerging embryonic part of the placenta. The comparison of protein expression and SCF complex activity showed that protein abundance does not correlate with the complex activity. At the MII stage, proteins were expressed at a low level, but the SCF complex activity was the highest. However, this does not mean that the level of protein expression does not influence ubiquitination by the SCF complex, as was shown by Piva and coauthors [62]. Altogether, our data suggest that SCF complex mediated protein degradation plays an important role in EGA initiation in bovines.

## Supporting Information

S1 Fig Relative mRNA expression of invariant members of SCF complex, embryos treated with α-amanitin, supplement. The data were normalised according to the relative concentration of the external standard (luciferase mRNA, 1pg per embryo). (A) *Cul1*, (B) *Skp1*, (C) *Rbx1*. Bars show ± S.D. ^a,b^ Values with different superscripts indicate statistical significance (P<0.05). (C, control group of untreated embryos; AA, group of embryos treated with α-amanitin; 2c, two-cell stage embryo; 4c, four-cell stage embryo; E8c, early eight-stage embryo; L8c, late eight-cell stage embryo).
(TIFF)

S2 Fig Western blot analysis of bovine oocytes and preimplantation embryos using anti-SKP1 antibody 1H9. 30 embryos per lane. A) Quantification of protein level. The data were processed using Quantity One software (Bio-Rad). 100% represents the sum of the trace quantities of all bands; relative abundance (y-axis) represents the percentage of each band. Bars show mean ± S.D. ^a,b^Values with different superscripts indicate statistical significance (P<0.05). The experiment was repeated four times, and a representative western blot image is shown below the graph. B) Representative image of additional bands (approximate size 55 and 90 kDa). (MII, MII oocytes; L8c, late eight-cell-stage embryos; 4c, four-cell-stage embryos).
(TIFF)

S3 Fig Confocal laser scanning microscopy of SKP1 (antibody 1H9) from MII oocytes to blastocyst-stage embryos. The embryos were labelled with mouse monoclonal anti-SKP1 antibody 1H9 (A’—F’) and the nuclei were stained with DAPI (A–F). In overlaid images (A”–F”), SKP1 is red and DNA blue.
(TIF)

S4 Fig Western blot analysis of SKP1 using Abnova antibody. First band shows SKP1 on zona pellucida, second band shows SKP1 in embryos.
(TIF)
