# Supplementary figures and images for: Characterization of SCF-Complex during Bovine Preimplantation Development
Source: PLoS One. 2016 Jan 29;11(1):e0147096. doi: 10.1371/journal.pone.0147096 (PMC4732672; doi:10.1371/journal.pone.0147096)

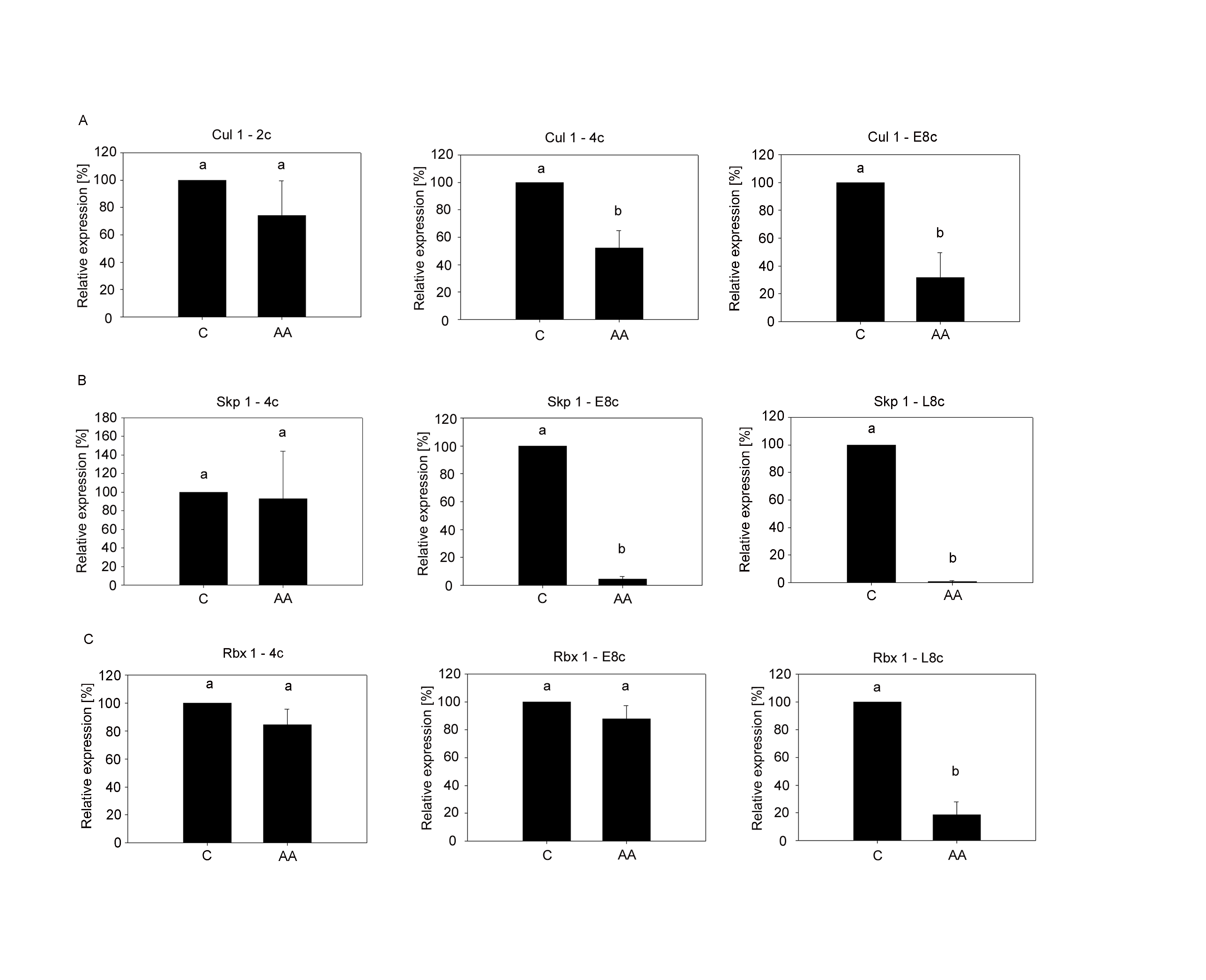

Supplement: S1 Fig — The data were normalised according to the relative concentration of the external standard (luciferase mRNA, 1pg per embryo). (A) Cul1, (B) Skp1, (C) Rbx1. Bars show ± S.D. a,b Values with different superscripts indicate statistical significance (P<0.05). (C, control group of untreated embryos; AA, group of embryos treated with α-amanitin; 2c, two-cell stage embryo; 4c, four-cell stage embryo; E8c, early eight-stage embryo; L8c, late eight-cell stage embryo). (TIFF) [file pone.0147096.s001.tiff]

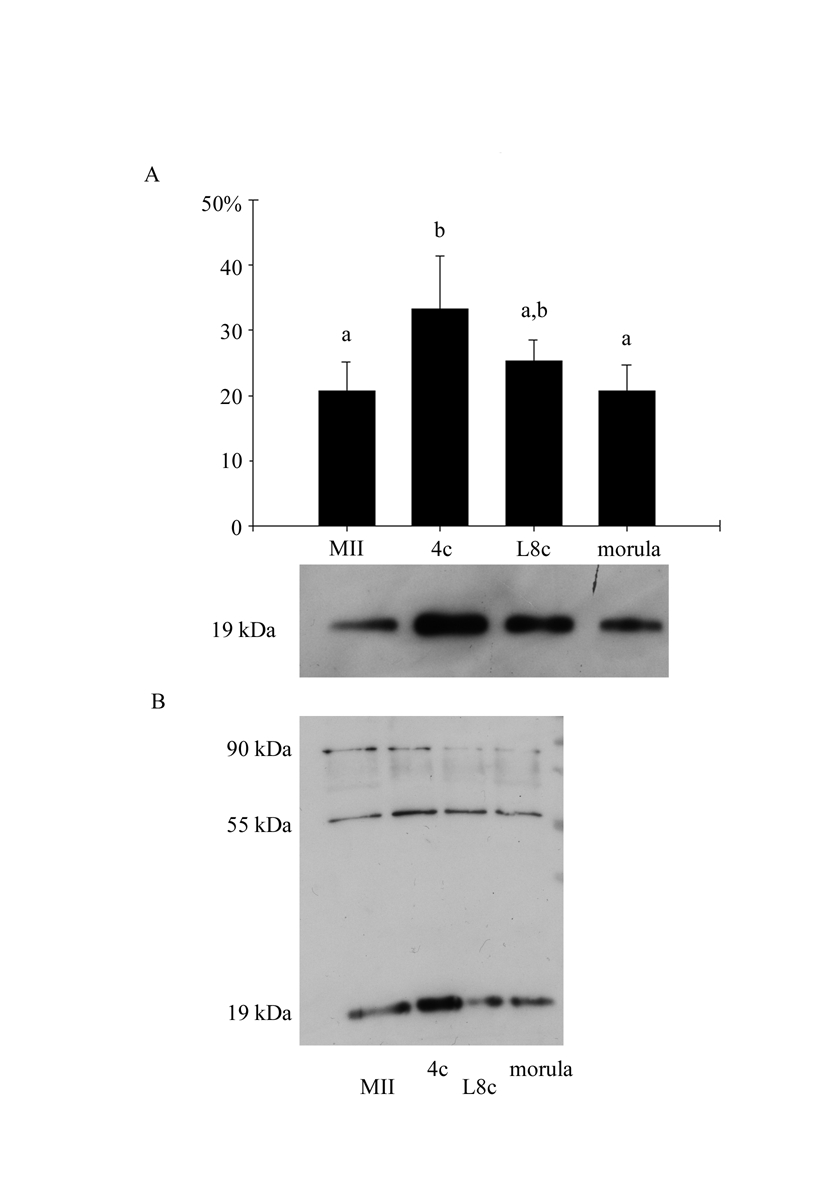

Supplement: S2 Fig — 30 embryos per lane. A) Quantification of protein level. The data were processed using Quantity One software (Bio-Rad). 100% represents the sum of the trace quantities of all bands; relative abundance (y-axis) represents the percentage of each band. Bars show mean ± S.D. a,bValues with different superscripts indicate statistical significance (P<0.05). The experiment was repeated four times, and a representative western blot image is shown below the graph. B) Representative image of additional bands (approximate size 55 and 90 kDa). (MII, MII oocytes; L8c, late eight-cell-stage embryos; 4c, four-cell-stage embryos). (TIFF) [file pone.0147096.s002.tiff]

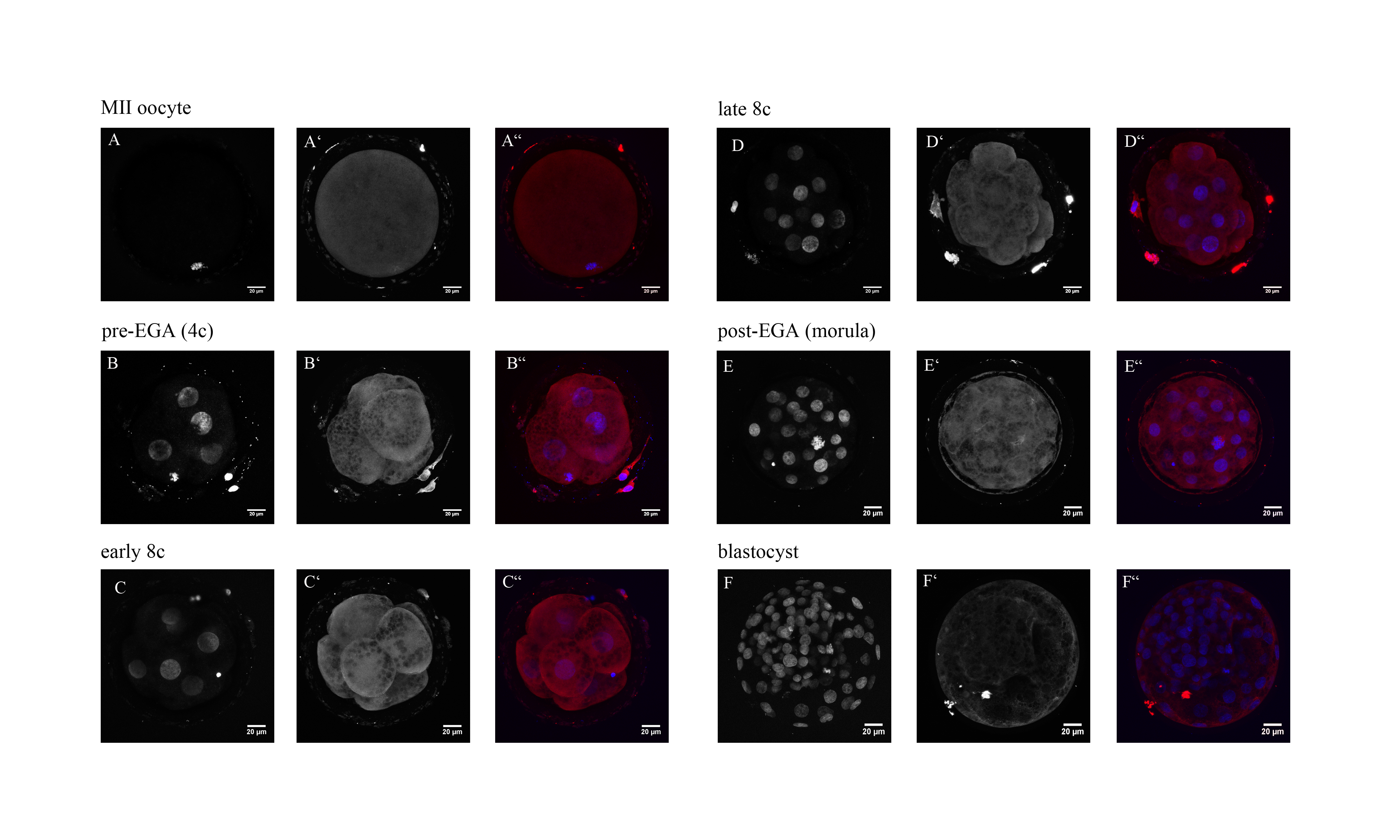

Supplement: S3 Fig — The embryos were labelled with mouse monoclonal anti-SKP1 antibody 1H9 (A’—F’) and the nuclei were stained with DAPI (A–F). In overlaid images (A”–F”), SKP1 is red and DNA blue. (TIF) [file pone.0147096.s003.tif]

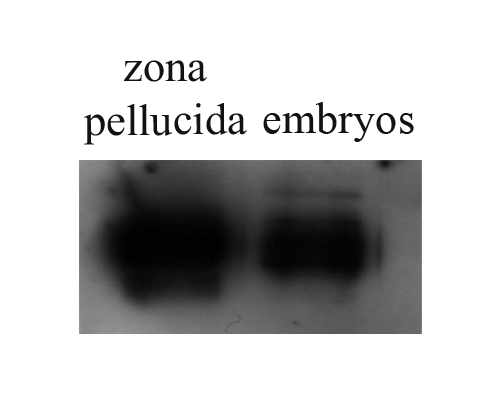

Supplement: S4 Fig — First band shows SKP1 on zona pellucida, second band shows SKP1 in embryos. (TIF) [file pone.0147096.s004.tif]
